# SHANK3 in vagal sensory neurons regulates body temperature, systemic inflammation, and sepsis

**DOI:** 10.3389/fimmu.2023.1124356

**Published:** 2023-02-08

**Authors:** Linlin Zhang, Sangsu Bang, Qianru He, Megumi Matsuda, Xin Luo, Yong-Hui Jiang, Ru-Rong Ji

**Affiliations:** ^1^ Center for Translational Pain Medicine, Department of Anesthesiology, Duke University Medical Center, Durham, NC, United States; ^2^ Department of Genetics, Pediatrics and Neuroscience, Yale University School of Medicine, New Haven, CT, United States; ^3^ Department of Neurobiology, Duke University Medical Center, Durham, NC, United States; ^4^ Department of Cell Biology, Duke University Medical Center, Durham, NC, United States

**Keywords:** autism, nodose ganglion, sepsis, TRPM2, TRPV1, vagus nerve, pain, inflammation

## Abstract

Excessive inflammation has been implicated in autism spectrum disorder (ASD), but the underlying mechanisms have not been fully studied. SHANK3 is a synaptic scaffolding protein and mutations of *SHANK3* are involved in ASD. Shank3 expression in dorsal root ganglion sensory neurons also regulates heat pain and touch. However, the role of Shank3 in the vagus system remains unknown. We induced systemic inflammation by lipopolysaccharide (LPS) and measured body temperature and serum IL-6 levels in mice. We found that homozygous and heterozygous *Shank3* deficiency, but not *Shank2* and *Trpv1* deficiency, aggravates hypothermia, systemic inflammation (serum IL-6 levels), and sepsis mortality in mice, induced by lipopolysaccharide (LPS). Furthermore, these deficits can be recapitulated by specific deletion of *Shank3* in Nav1.8-expressing sensory neurons in conditional knockout (CKO) mice or by selective knockdown of *Shank3* or *Trpm2* in vagal sensory neurons in nodose ganglion (NG). Mice with *Shank3* deficiency have normal basal core temperature but fail to adjust body temperature after perturbations with lower or higher body temperatures or auricular vagus nerve stimulation. *In situ* hybridization with RNAscope revealed that *Shank3* is broadly expressed by vagal sensory neurons and this expression was largely lost in *Shank3* cKO mice. Mechanistically, *Shank3* regulates the expression of *Trpm2* in NG, as *Trpm2* but not *Trpv1* mRNA levels in NG were significantly reduced in *Shank3* KO mice. Our findings demonstrated a novel molecular mechanism by which Shank3 in vagal sensory neurons regulates body temperature, inflammation, and sepsis. We also provided new insights into inflammation dysregulation in ASD.

## Introduction

Autism spectrum disorder (ASD) is defined by persistent deficits in the ability to initiate and to sustain reciprocal social interaction and social communication and characterized by a range of restricted, repetitive, and inflexible patterns of behavior, as well as various sensory abnormalities (1–5). SHANK3 is a scaffolding protein located at excitatory synapses, and mutations of *SHANK3* (haploinsufficiency) may account for 1-2% of all ASD cases including Phelan-McDermid syndrome (PMS) (2, 6, 7). The disrupted striatum centered brain structures and synaptic transmission in this brain region are associated with ASD children with *SHANK3* mutations and animal models of *Shank3* deficiency (2, 4, 8, 9).

Accumulating evidence has indicated the link between ASD and generalized immune dysfunction (hyper-inflammation). For example, proinflammatory cytokines (e.g., TNF-α, IL-6, IL-8) are increased in the brains of ASD individuals (10). Peripheral blood mononuclear cells from ASD patients produced more TNF-α, IL-1β, and IL-6 than control cells (11). The plasma and serum concentrations of IL-1β, IL-6, and IL-8 are also elevated in ASD samples compared with the healthy control samples (12). Upregulations of IL‐6 and IL‐17 levels were reported in pregnant rodent mothers upon maternal immune activation and IL‐6 may induce the secretion of IL‐17 (3). IL-6 and IL-17 were shown to promote autistic behaviors in animal models (3, 13–15). By contrast, anti-IL-6 or anti-IL-17 treatments can alleviate the autistic behaviors induced by maternal immune activation (3, 14). Frequent respiratory infections were found in 57% of ASD patients and regarded as an ASD comorbidity (4). Anti-biotic treatment may improve ASD-related behaviors (16–18). Subacute neuropsychiatric syndrome in girls with *SHANK3* mutations responds to immunomodulation (19). Preclinical study shows that acute inflammatory challenge induced by lipopolysaccharide (LPS) can unmask behavioral deficits of *Shank3* ^+/−^ mice (7). However, the mechanisms of excessive immune reaction in ASD are not fully understood.

Emerging roles for the gut microbiome and gut-brain axis have been implicated in ASD (18, 20, 21). Notably, the vagus system plays a critical role in regulating the gut-brain axis (18, 22, 23). Precision microbial-based therapy was shown to rescue social deficits in mouse models of ASD and, interestingly this rescue in *Shank3B* knockout mice depends upon the function of the vagus nerve (20). The molecular mechanisms underlying the vagus nerve modulation of ASD remain largely unknown. Recent studies have shown SHANK3 expression in the peripheral nervous system, such as primary sensory neurons of dorsal root ganglion (DRG); and furthermore, SHANK3 interacts with transient receptor potential ion channel V1 (TRPV1) and GABA_A_ receptor in DRG neurons to regulate pain (24) and touch (25). Here we show that *Shank3* is also expressed by vagal sensory neurons and *Shank3* in nodose ganglion (NG) sensory neurons plays a crucial role in maintaining body temperature and protecting against systemic inflammation in responses to LPS challenge and temperature perturbations. Mechanistically, SHANK3 regulates the expression of TRPM2 (transient receptor potential melastatin 2), a nonselective Ca^2+^ permeable cation channel in NG vagal sensory neurons.

## Materials and methods

### Regents

AAV.CMV.HI. eGFP-Cre.WPRE.SV40 was from Addgene (Plasmid #105545). Shank3 siRNA (Cat# AM16708, ID:194441), Trpv1 siRNA (# AM16708, ID: 223137), and Trpm2 siRNA (# 1320001, ID: mss219854) were purchased from Thermo scientific. Control siRNA was purchased from Santa cruz biotech (#sc-37007), and RVG peptide (# AS-62566) was from Anaspec (Fremont, CA). Menthol (# W266507), capsaicin (# M2028), and LPS (# L2630) were purchased from Sigma.

### Animals

*Shank3* knockout mice (exons 4 to 22 deletion, Δ4–22, C57BL/6 background RRID: MGI:5800311) (8), Shank3 flox/flox mice (RRID: MGI: 5800310) (26), and Shank2 flox/flox mice were generated at Yong-Hui Jiang’s laboratory at Duke University Medical Center. Both Shank2 and Shank3 floxed mice were backcrossed to C57BL/6J for more than 10 generations. To generate sensory neuron-specific deletion of Shank3 and Shank2, the flox mice were crossed with Nav1.8-cre mice, provided by Dr. Rohini Kuner of University of Heidelberg (27). Adult mice (males and females, 8–10 weeks) were used for behavioral and biochemical studies. CD1 mice (males and females, 8–10 weeks, Charles River Laboratories) were used for some siRNA knockdown experiments. All the mouse procedures were approved by the Institutional Animal Care & Use Committee of Duke University. Two to five mice were housed in each cage under a 12-hour alternating light-dark cycle with ad libitum access to water and food. Sample sizes were estimated based on our previous studies for similar types of behavioral and biochemical analyses (24, 28).

### Peri-neural injection of virus or siRNA in the vagus nerve

The siRNA preparation and injection were conducted as we previously described (29). For siRNA delivery to the vagus nerve, we mixed 100 nM siRNA with 1 µM RVG peptide that can facilitate siRNA uptake by neurons (30). For AAV9 virus delivery to vagus nerve, we mixed 1 x 10^9^ virus in 10 µl of PBS. The vagal nerve was exposed as previously described (31). Mice were anesthetized with isoflurane and a midline cervical muscle and salivary glands were exposed by blunt dissection until the trachea was visible. The vagus nerve was gently separated from the artery, and bilateral peri-neural injections were conducted to deliver siRNA or virus (5 μl) using a glass electrode with tip size of 1 µm. The knockdown effects of siRNA and AAV9 virus were validated by examining the expression of *Shank3*, *Trpm2*, and *Trpv1* in vagal sensory neurons in nodose ganglion (NG) using *in situ* hybridization.

### Vagal nerve stimulation

The 5% menthol and 100 µM capsaicin were painted into left ear auricular acupoint or skin (50 µl) to measure the body temperature change. Mice were lightly anesthetized with 2.5% isoflurane and then painted by fine brush.

### LPS model of systemic inflammation and rectal temperature measurement

The systemic inflammation was generated by intraperitoneal injection of 1 mg/kg LPS (from Escherichia coli O111:B4) prepared in PBS (100 mg/ml stock). Body temperature measured before LPS injection, then at 0.5, 1, 3, 6, 24, 48, 72, and 96 hours after the LPS injection. The environmental temperature was maintained at 23 ± 0.5°C. To obtain a rectal temperature, the mouse is hand-restrained (briefly anesthetized) and placed on a horizontal surface (cage lid) with the tail lifted, and a probe (covered with Vaseline) was gently inserted into the rectum to a fixed depth of 2 cm. The rectal temperature was measured by the TA-29 flexible implantable probe and TC-344C temperature measuring unit (Warner Instruments., Hamden, CT, USA).

### Electrocardiogram recordings

For ECG recording, the silver wires were implanted in the left abdomen below the heart and the right upper shoulder. Two electrodes were fixed to the incised tissues in mice using a 26 G needle under isoflurane anesthesia (32). The silver wires were exposed outside the neck region and put on the small jacket for the prevention of biting. The silver wires were exposed outside the neck region for the prevention of biting. After exposure to the menthol into ear acupoint region (concha area in outer ear), the ECG activity was measured in awake mice. The shaking induced electrical noise was excluded during data analysis. The analog electrical signal was amplified by a microelectrode ac amplifier (AM systems, model 1800) and converted by Digidata 1440A (Molecular device). Signals were filtered at 2 kHz and digitized at 5 kHz. Data were stored and analyzed with a personal computer using pCLAMP 10 (Molecular Devices) software.

### RNAscope *in situ* hybridization

Mice were briefly anesthetized with 4% isoflurane and transcardially with PBS and 4% paraformaldehyde. Following perfusion, nodose ganglia (NG) were collected and postfixed in the same fixative overnight at 4°C. NG tissues were embedded in OCT medium (Tissue-Tek), sectioned (14 μm) in a cryostat, and thaw-mounted onto Superfrost Plus slides (VWR). *In situ* hybridization was performed using the RNAscope system (Advanced Cell Diagnostics) following the manufacturer’s instructions. The RNA scope probes for mouse *Shank3* (# 417371), *Trpm2* (# 316831-C3), and *Trpv1* (# 313331-C3) were designed by Advanced Cell Diagnostics and the RNAscope multiplex fluorescent assays were performed according to the manufacturer’s instructions. Pre-hybridization and hybridization, and then washing were performed according to standard methods. All images were acquired with the same settings using a confocal microscope under 20x magnification. For quantification, four non-adjacent NG sections from each animal and four animals per group were included. The intensity score (0: no signal, 1: weak signal; 2: medium signal, and 3: strong signal) was used to semi-quantify the mRNA signals blindly. We also quantified the number of mRNA-labeled punctate dots (puncta) in each neuron using Image J.

### RT-qPCR

Total RNAs from nodose ganglia were extracted using the Direct-zol™ RNA MiniPrep Kit (Zymo Research), and 0.5-1 μg of RNA was reverse-transcribed using the iScript cDNA Synthesis^®^ (Bio-Rad). Specific primers, including *Gapdh* control, were designed using IDT SciTools Real-Time PCR software. We performed gene-specific mRNA analyses using the CFX96 Real-Time PCR system (BioRad). Quantitative PCR amplification reactions contained the same amount of Reverse transcription (RT) product, including 10 μl of 2× iQSYBR-green mix (BioRad) and 100-300 nM of forward and reverse primers in a final volume of 15 μl. Primer efficiency was obtained from the standard curve and integrated for calculation of the relative gene expression, which was based on real-time PCR threshold values of different transcripts and groups.

*Trpm2* forward: TGCCTCACCTGCTCTTTGCC

*Trpm2* reverse: TCTGTGTGTTCCTGCACCTA

*TRPV1* forward: ATGTTCGTCTACCTCGTGTTCTTG

*TRPV1* reverse: AGGCAGTGAGTTATTCTTCCCATCC

*TRPM8* forward GTTGGACCTTGCCAGTGATGAG

*TRPM8* reverse CCATTCTCCAGAAAGAGGCGGA

*SCN10A* forward ATGGAGGTCAGCCAGGACTACA

*SCN10A* reverse CTGTGAGGTTGTCCGCACTGAA

*Gapdh* forward: AGGTCGGTGTGAACGGATTTG

*Gapdh* reverse: GGGGTCGTTGATGGCAACA

### ELISA

To detect serum cytokine levels, we purchased mouse ELISA kits from R&D system (Minneapolis, MN, Cat# DY406 for IL-6). We collected whole blood from a facial vein (30 μl~60 μl). The collected blood was allowed to clot for 30 minutes at room temperature. The clot was then removed in a refrigerated centrifuge at 2,000 x g for 10 min to collect the supernatant (serum). To perform each ELISA assay, we used 5 μl of serum. We measured the absorbance of OD450 using a microplate reader (Bio-Rad) and then quantified the data by MPM6 software (BioRad).

### Statistical analysis

All the data in the figures were expressed as mean ± SEM. We analyzed the biochemical or survival data using Prism GraphPad 6.0. One-Way ANOVA with Bonferroni *post hoc* or Two-Way ANOVA with Tukey’s posthoc test or unpaired t-test were used to assess biochemical, temperature, and ECG data. We analyzed the survival ratio using a Kaplan–Meier method and log-rank (Mantel-Cox) test. If the overall log-rank test was statistically significant (*p* < 0.05), we further performed pairwise comparisons of two groups. *p* < 0.05 was considered statistically significant.

## Results

### LPS-induced hypothermia, systemic inflammation and mortality are aggravated in *Shank3* global knockout mice

**Figure 1:**
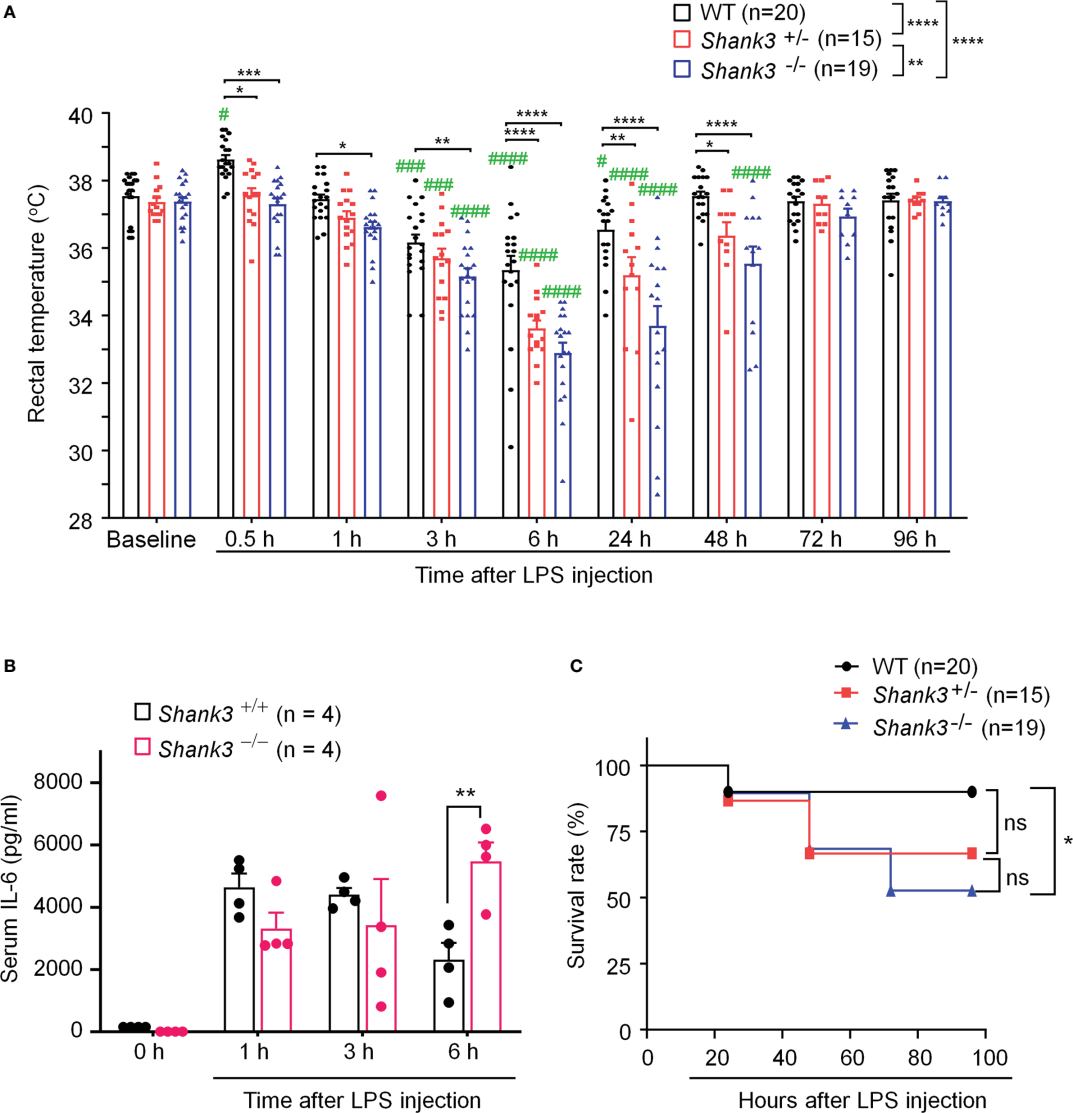
*Shank3* global mutants exhibit exacerbation in LPS-induced hypothermia, systemic inflammation and mortality in C57BL/6 mice. **(A)** Time course of rectal temperature after LPS injection in Wild-type (WT, *Shank3* ^+/+^) mice, heterozygous (*Shank3* ^+/−^) mice and homozygous (*Shank3* ^−/−^) mice. **(B)** Serum IL-6 levels, revealed by ELISA, in WT and *Shank3* ^−/−^ mice at different times with LPS. **(C)** Survival curves of WT, *Shank3* ^+/−^ and *Shank3* ^−/−^ mice treated with LPS. LPS (1mg/kg) was injected *via* intraperitoneal (i.p.) route. Data are expressed as mean ± SEM and analyzed by two-way ANOVA with Bonferroni *post hoc* test **(A, B)** and Mantel-Cox test **(C)**. ^#^ *p <*0.05, ^###^ *p <*0.001, ^####^ *p <*0.0001 versus baseline. ^*^ *p <*0.05, ^**^ *p <*0.01, ^***^ *p <*0.001, ^****^ *p <*0.0001 versus WT mice. Sample sizes are indicated in brackets. n.s., not significant.

To investigate an overall role of *Shank3* in LPS-induced hypothermia, inflammation, and mortality, we employed *Shank3* △e4-22 mutant mice, in which exons 4 to 22 were deleted and SHANK3 protein is completely lost (8). In the current study, we referred to △e4-22 mutant mice as *Shank3* global knockout mice (GKO). First, we compared the baseline body temperature in wild-type (WT) and heterozygous (*Shank3^+/-^*) and homozygous (*Shank3^-/-^*) GKO mice. The rectal temperature analysis revealed that *Shank3^-/-^* mice exhibited no change in body core temperature as compared to WT and *Shank3^+/-^* mice (**Figure 1A**), suggesting that SHANK3 is not involved in the regulation of basal body temperature. Then, we challenged these mice with intraperitoneal (i.p.) injection of LPS (1 mg/kg) to induce sepsis (28). Herein, we found a rapid and transient hyperthermia in WT mice, as indicated by an elevation in rectal temperature at 30 minutes after LPS injection as compared to baseline (*p* = 0.0158, **Figure 1A**). Interestingly, this hyperthermia was completely compromised in both *Shank3^+/-^* and *Shank3^-/-^* mice (*p* = 0.0106 and *p* = 0.0004, respectively) when compared with WT mice (**Figure 1A**). We also revealed that LPS-induced hypothermia started at 3 hours (*p* = 0.0008), peaked at 6 hours (*p* < 0.0001), and continued for 24 hours (p=0.0488) after LPS intervention in WT mice. Furthermore, as compared to WT mice, this hypothermia was observed within 1 hour (*p* = 0.0411) and lasted for at least 48 hours (p < 0.0001) after LPS exposure in *Shank3^-/-^* mice. More strikingly, sepsis-induced hypothermia was significantly aggravated in both *Shank3^+/-^* and *Shank3^-/-^* mice (F (2, 408) = 48.24, *p* < 0.0001, two-way ANOVA, **Figure 1A**). There was also significant difference between heterozygous and homozygous mice (F (1, 245) = 11.35, *p* = 0.0011, two-way ANOVA, **Figure 1A**). This result suggested that loss of one copy of *Shank3* is sufficient to cause hyperthermia defects and hypothermia exacerbation, which is reminiscent of autism patients with haploinsufficiency of *SHANK3*.

Next, we investigated whether sepsis-induced systemic inflammation was altered in *Shank3* mutant mice. Pro-inflammatory cytokine IL-6 is well recognized as a biomarker for sepsis and sepsis-associated death (28). We detected robust increases of serum IL-6 levels at 6 hours after LPS injection in *Shank3^-/-^* mice as compared to WT mice (F (1, 24) = 0.1450, *p* = 0.008226, two-way ANOVA, **Figure 1B**).

Also, we evaluated survival rate following LPS injection among three genotypes. Low-dose LPS at 1 mg/kg resulted in a very low mortality (10%, **Figure 1C**) in WT mice. As compared to WT mice, we found a slight increase (23.33%) in mortality in LPS-treated *Shank3^+/-^* mice (*p* = 0.1062, **Figure 1C**) but a significant increase (37.37%) in mortality in LPS-treated *Shank3^-/-^* mice (*p* = 0.0152, **Figure 1C**). Collectively, these data indicate that SHANK3 confers protection against hypothermia, systemic inflammation and septic death following LPS injection.

### *Shank3* deletion in vagal sensory neurons exacerbates LPS-induced hypothermia, systemic inflammation, and mortality

**Figure 2:**
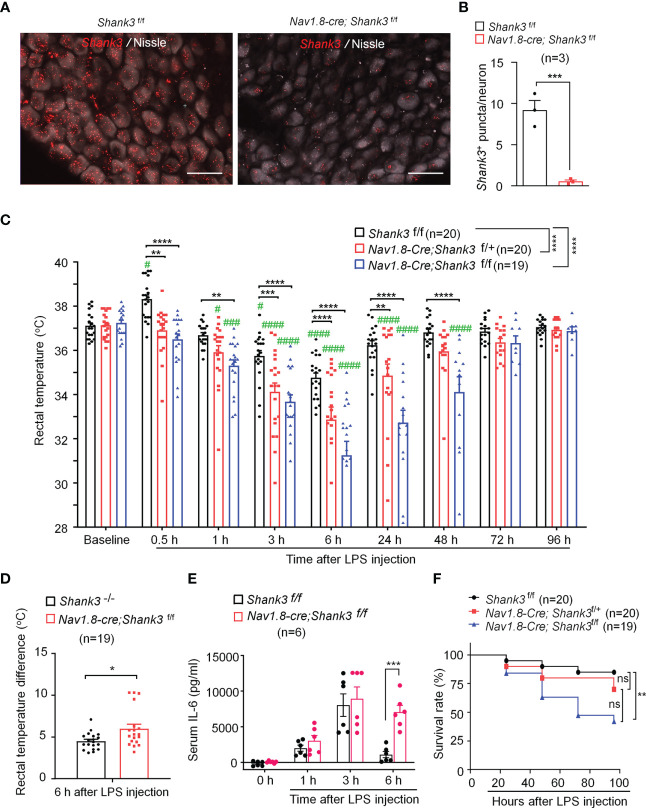
LPS-induced hypothermia, systemic inflammation and mortality are aggravated in *Shank3* CKO C57BL/6 mice. *Shank3* CKO mice were generated by crossing *Shank3*-floxed mice with *Nav1.8*-*Cre* mice, leading to specific loss of *Shank3* in Nav1.8-expressing sensory neurons. **(A)** *In situ* hybridization (ISH) images showing *Shank3* mRNA (red) expression in vagal sensory neurons in nodose ganglia (NG) of *Shank3* ^f/f^ mice and *Nav1.8-cre*; *Shank3* ^f/f^ mice. Scale bar, 50 μm. **(B)** Quantification of the number of *Shank3* puncta in NG neurons. **(C)** Time course of rectal temperature after LPS (1mg/kg, i.p.) injection in *Shank3* ^f/f^ mice, *Nav1.8-cre*; *Shank3* ^f/+^ mice and *Nav1.8-cre*; *Shank3* ^f/f^ mice. **(D)** Changes (reduction) of rectal temperature at 6 hours following LPS injection in GKO (*Shank3* ^−/−^) mice and CKO (*Nav1.8-cre*; *Shank3* ^f/f^) mice. **(E)** Serum IL-6 levels in *Shank3* ^f/f^ mice and *Nav1.8-cre*; *Shank3* ^f/f^ mice treated with LPS. **(F)** Survival curves of three genotypes mice treated with LPS. Data are expressed as mean ± SEM and analyzed by unpaired two-tailed *t* test **(B, D)**, two-way ANOVA with Bonferroni *post hoc* test **(C, E)**, and Mantel-Cox test **(F)**. ^#^ *p* < 0.05, ^###^ *p* < 0.001, ^####^ *p* < 0.0001 versus baseline. ^*^ *p* < 0.05, ^**^ *p* < 0.01, ^***^ *p* < 0.001, ^****^ *p* < 0.0001 versus *Shank3* ^f/f^ mice. Sample sizes are indicated in brackets. n.s., not significant.

Vagus nerve stimulation has been shown to protect against sepsis and systemic inflammation (22). Recent studies reported *Shank3* expression in peripheral sensory neurons of DRG (24, 25). However, the expression and role of SHANK3 in the vagus system is unclear. Using a sensitive RNA scope assay, we found extensive *Shank3* mRNA expression in majority of vagal sensory neurons in nodose ganglia (NG, **Figure 2A**). To evaluate an underlying contribution of peripheral SHANK3 to hypothermia regulation, we generated *Shank3* conditional knockout mice by crossing *Shank3*-floxed mice with *Nav1.8*-*Cre* mice, leading to specific deletion of *Shank3* in *Nav1.8-*expressing peripheral sensory neurons including NG neurons (33). In this study, we referred to *Shank3^f/f^* mice as littermate control wildtype (WT) mice, and referred to *Nav1.8-cre; Shank3^f/+^* and *Nav1.8-cre; Shank3^f/f^* mice as *Shank3* conditional knockout (CKO) heterozygous and homozygous mice. Notably, *Shank3* expression was substantially reduced in CKO mice (*Nav1.8-cre; Shank3^f/f^* mice) as compared to *Shank3^f/f^* mice (*p* = 0.0017, **Figures 2A, B**). This result indicates a selective expression of *Shank3* in NG sensory neurons and also validated *Shank3* CKO mice.

As in GKO mice, body temperature analysis revealed no difference of basal body temperature between WT and CKO mice (**Figure 2C**), suggesting that *Shank3* in sensory neurons does not contribute to basal body temperature. We next compared hypothermia phenotypes after LPS injection in three genotypes of WT and CKO mice. Herein, we reported a rapid and transient hyperthermia in *Shank3^f/f^* mice, as indicated by the elevation in rectal temperature at 30 minutes after LPS injection as compared to baseline (*p* = 0.0473, **Figure 2C**). Interestingly, this hyperthermia was completely compromised in both heterozygous and homozygous CKO mice (*p* = 0.0025 and *p* < 0.0001, respectively) when compared with *Shank3^f/f^* mice. Furthermore, homozygous CKO mice exhibited more rapid (within 1 hour, *p*=0.0038) and more lasting (>48 hours, *p* < 0.0001) hypothermia, as compared to *Shank3^f/f^* mice (**Figure 2C**), suggesting that *Shank3* deficiency in sensory neurons accelerates the induction of hypothermia and delays the resolution of hypothermia after sepsis. Intriguingly, sepsis-induced hypothermia was significantly aggravated in both heterozygous and homozygous CKO mice (F (2, 448) = 58.38, *p* < 0.0001, two-way ANOVA, **Figure 2C**). There was also significant difference between heterozygous and homozygous CKO mice (F (1, 286) = 16.47, *p* < 0.0001, two-way ANOVA, **Figure 2C**). More strikingly, *Shank3* CKO mice exhibited more remarkable changes (reductions) in rectal temperature when compared with *Shank3* GKO (*p* = 0.0136, **Figure 2D**), suggesting a dominant role of peripheral SHANK3 in temperature regulation.

Next, we investigated whether LPS-induced systemic inflammation would also be affected in *Shank3* CKO mice. Serum IL-6 levels did not differ at 3 hours and 6 hours after LPS stimulation in WT and CKO mice. At 24 hours, IL-6 levels returned to baseline in WT mice but remained at the peak in CKO (F (3, 40) = 29.21, *p* = 0.0002, two-way ANOVA, **Figure 2E**). Survival rate analysis showed that LPS significantly increased mortality (42.90%) in homozygous CKO mice (p=0.0083, vs. WT, **Figure 2F**). Collectively, these data demonstrate that SHANK3 in *Nav1.8-*expressing peripheral sensory neurons confers protection against hypothermia, systemic inflammation and septic death after sepsis.

### SHANK3 in peripheral vagal sensory neurons is required for maintaining the homeostasis of core temperature

**Figure 3:**
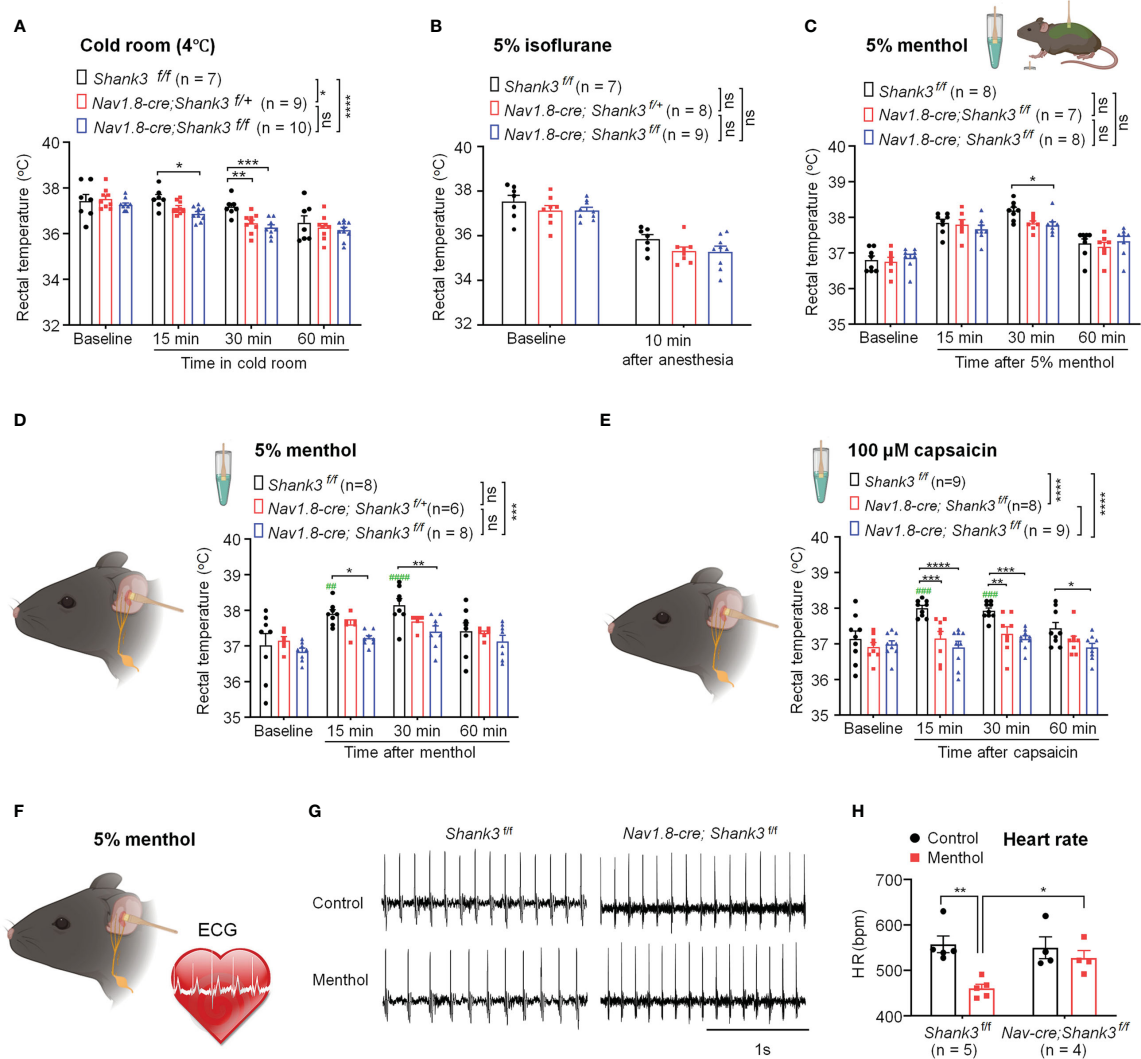
*Shank3* in sensory neurons is required for the maintenance of body temperature after perturbations in C57BL/6 mice. **(A)** Time course of rectal temperature after acute exposure to a cold room (4°C) in three genotypes. **(B)** Time course of rectal temperature after brief anesthesia to 5% isoflurane in three genotypes. **(C)** Time course of rectal temperature after 5% menthol smearing on body trunk in *Shank3* ^f/f^ mice, *Nav1.8-cre*; *Shank3* ^f/+^ mice and *Nav1.8-cre*; *Shank3* ^f/f^ mice. **(D)** Time course of rectal temperature after auricular painting of 5% menthol in three genotypes. **(E)** Time course of rectal temperature after auricular painting of 100 μM capsaicin in three genotypes. **(F)** Experimental design for ECG recording after auricular painting of 5% menthol in littermate control WT and CKO mice. **(G)** Representative traces of ECG in *Shank3* ^f/f^ mice and *Nav1.8-cre*; *Shank3* ^f/f^ mice following vehicle or menthol treatment. **(H)** Heart rate (HR) in *Shank3* ^f/f^ mice and *Nav1.8-cre*; *Shank3* ^f/f^ mice following vehicle or menthol treatment. Data are expressed as mean ± SEM and analyzed by two-way or one-way ANOVA with Bonferroni *post hoc* comparisons. ^##^ *p* < 0.01, ^###^ *p* < 0.001, ^####^p < 0.0001, ^####^p < 0.0001, versus baseline. ^*^ *p* < 0.05, ^**^ *p* < 0.01, ^***^ *p* < 0.001, ^****^ *p* < 0.0001 versus *Shank3* ^f/f^ mice. Sample sizes are indicated in brackets. n.s., not significant.

To investigate a specific role of peripheral SHANK3 in regulating body temperature, we challenged WT and *Shank3* CKO mice in different conditions of hypothermia and hyperthermia, including cold room habituation at 4°C (**Figure 3A**), 5% isoflurane anesthesia (**Figure 3B**), intrathecal injection of PGE_2_ (200 ng, **Figure S3**), 5% menthol application to the back and auricular region (**Figures 3C, D**), and auricular application of 100 μM capsaicin (**Figure 3E**). Cold room testing showed deficits in homozygous CKO mice (F (1, 60) = 19.49, *p* < 0.0001, two-way ANOVA, vs. WT) and heterozygous CKO mice (F (1, 56) = 5.568, *p* = 0.0168 vs. WT) in maintaining core temperature (**Figure 3A**). Interestingly, CKO mice exhibited rapid hypothermia compared to WT mice (F (2, 92) = 10.54, *p* < 0.05 vs. WT, **Figure 3A**). Brief anesthesia to 5% isoflurane produced a rapid drop in body temperature in 10 min, but no difference was found between three genotypes (F (2, 42) = 3.094, *p* = 0.0558, two-way ANOVA, **Figure 3B**). Intrathecal PGE_2_ induced hyperthermia and fever in WT mice, which was impaired in *Shank3* CKO mice (F (2, 48) = 3.835, *p* = 0.0285, two-way ANOVA, **Figure S1**). Next, we observed a rapid and transient hyperthermia after 5% menthol smearing in body trunk in *Shank3^f/f^* mice (**Figure 3C**). This hyperthermia at 30 min was partially attenuated in homozygous CKO mice (*p* = 0.0218, vs. WT), although we did not observe any significant difference among three genotypes (F (2, 80) = 1.776, *p* = 0.176, two-way ANOVA, **Figure 3C**). Next, we smeared 5% menthol to auricular vagal region to mimic vagus nerve stimulation (aVNS, **Figure 3D**). This cold/cool stimulation induced hyperthermia in WT mice but not in homozygous CKO mice (F (1, 56) = 12.85, *p* = 0.0007, two-way ANOVA, **Figure 3D**), which was consistent with the results in *Shank3* GKO mice (**Figure S2A**). Similarly, aVNS with 100 μM capsaicin smearing produced hyperthermia in *Shank3^f/f^* mice, which was compromised in both heterozygous and homozygous CKO mice (F (2, 92) = 22.51, *p* < 0.0001, two-way ANOVA, **Figure 3E**). The same result was also observed in *Shank3* GKO mice (**Figure S2B**).

Next, we recorded electrocardiogram (ECG) to assess VNS-induced heart rate (HR) change following auricular menthol smearing (**Figure 3F**). As expected, menthol-induced VNS resulted in a significant decrease of HR in WT *Shank3^f/f^* mice, which was abolished in homozygous CKO mice (F (1, 14) = 11.73, *p* = 0.0041, two-way ANOVA, **Figures 3G, H**). Collectively, these detailed results suggest that peripheral *Shank3* in vagal sensory nerve plays an important role in maintaining core temperature following perturbations.

### Selective SHANK3 knockdown in vagal sensory neurons exacerbates LPS-induced hypothermia, systemic inflammation and mortality

**Figure 4:**
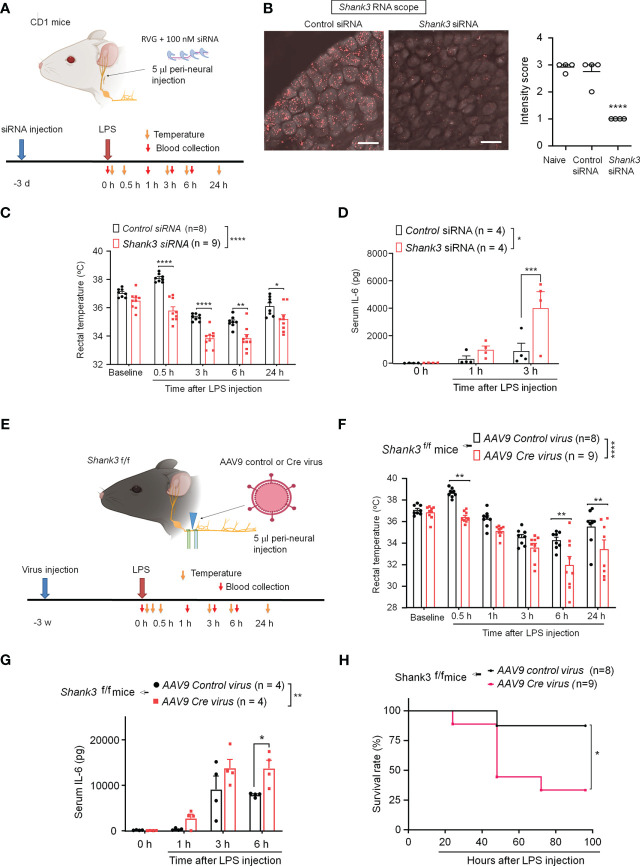
SHANK3 knockdown in vagal sensory neurons aggravates LPS-induced hypothermia, systemic inflammation and mortality in CD1 mice **(A–D)** and C57BL/6 mice **(E–G)**. **(A)** Experimental design for SHANK3 knockdown, LPS injection, body temperature measurement and blood collection in CD1 mice. Peri-neural injection of 100 nM *Shank3* siRNA (5 μl, mixed with RVG peptide, 1:10) was made to knockdown SHANK3 expression in vagal sensory neurons in NG. **(B)** ISH images showing *Shank3* mRNA (red) expression in NG following control siRNA and *Shank3* siRNA treatment. Scale bar, 50 μm. **(C)** Time course of rectal temperature after LPS injection (1mg/kg, i.p.) in control siRNA and *Shank3* siRNA treated mice. **(D)** Serum IL-6 levels after LPS exposure in control siRNA and *Shank3* siRNA treated mice. **(E)** Experimental design for SHANK3 knockdown, LPS injection, body temperature measurement and blood collection in *Shank3* ^f/f^ mice. Peri-neural AAV9-Cre virus injection was made to knockdown SHANK3 expression in vagal sensory neurons in NG in *Shank3* ^f/f^ mice. **(F)** Time course of rectal temperature after LPS (1mg/kg) injection in AAV9 control virus and AAV9 *Cre* virus treated mice. **(G)** Serum IL-6 levels and **(H)** Survival curves after LPS exposure in AAV9 control virus and AAV9 *Cre* virus treated mice. Data are expressed as mean ± SEM and analyzed by one-way ANOVA with Bonferroni *post hoc* test **(B)**, two-way ANOVA with Bonferroni *post hoc* test **(C, D, F, G)**, and Mantel-Cox test **(H)**. ^*^ *p* < 0.05, ^**^ *p* < 0.01, ^***^ *p* < 0.001, ^****^ *p* < 0.0001 versus mice treated control siRNA or AAV9 control virus. Sample sizes are indicated in brackets. n.s., not significant.

SHANK3 is expressed by different types of peripheral sensory neurons in mouse DRG, NG, and trigeminal ganglia (34). To confirm a specific role of peripheral SHANK3 in vagal nerve for regulating the LPS-induced effects, we used *Shank3* siRNA to knockdown endogenous SHANK3 expression in vagal sensory neurons in NG. A short peptide derived from rabies virus glycoprotein (RVG) has been utilized to facilitate siRNA uptake by neurons including sensory neurons and enhance knockdown efficiency of siRNA (29, 30). We performed bilateral peri-vagal nerve delivery of *Shank3* siRNA (mixed with the RVG peptide, 1:10) in CD1 mice (**Figure 4A**). *In situ* hybridization (ISH) revealed a profound reduction in *Shank3* mRNA in NG neurons following *Shank3* siRNA (100 nM) treatment (F (2, 9) = 48.70, *p* = 0.0002, one-way ANOVA, **Figure 4B**), suggesting the successful establishment of SHANK3 knockdown by siRNA. We next challenged these mice with LPS on 3 days following siRNA injection. Intriguingly, we found that LPS-induced transient hyperthermia in control siRNA treated mice was completely lost after *Shank3* siRNA treatment (*p* < 0.0001, **Figure 4C**). Moreover, LPS-induced hypothermia was significantly aggravated by vagal SHANK3 knockdown (F (1, 75) = 90.52, *p* < 0.0001, two-way ANOVA, vs. control siRNA, **Figure 4C**). Peri-vagal *Shank3* siRNA (50 nM, 100 nM and 200 nM) enhanced LPS-induced hypothermia in a dose-dependent manner (F (5, 297) = 53.45, *p* < 0.0001, two-way ANOVA, **Figure S3A**). Furthermore, we detected a robust increase of serum IL-6 levels at 3 hours after LPS injection (*p* = 0.0027, *Shank3* siRNA vs. control siRNA, **Figure 4D**). However, we did not detect any difference of mortality between control siRNA and *Shank3* siRNA treatment, as CD1 mice are more resistant to sepsis than C57BL/6 mice (**Figures S3B–D**).

Next, we employed GFP fluorescence-labeled AAV9-*Cre* virus to produce a more persistent knockdown of endogenous SHANK3 expression in vagal sensory neurons, by using bilateral peri-vagal nerve delivery of AAV9 *Cre* virus in *Shank3^f/f^* mice (**Figure 4E**). Peri-vagal nerve injection of GFP-labeled AAV9 *Cre* virus resulted in uptake of AAV9 *Cre* virus in NG neurons as a result of axonal transport from the vagal nerve (**Figure S4A**). Simultaneously, we detected a profound reduction in *Shank3* mRNA in NG neurons following the same treatment (*p* = 0.0002, **Figures S4A–C**), manifesting the successful establishment of SHANK3 knockdown by *Cre* virus in *Shank3^f/f^* mice. We next challenged these mice with LPS at 2 weeks following the virus injection. We found that the *Cre* virus treatment abolished the LPS-induced transient hyperthermia (*p* = 0.002 vs. control virus) and exacerbated the LPS-induced hypothermia (F (1, 89) = 36.12, *p* < 0.0001, two-way ANOVA, vs. control virus, **Figure 4F**). Moreover, the *Shank3* virus treated mice exhibited a significant increase of serum IL-6 levels (F (1, 24) = 10.18, *p* = 0.0039, Two-Way ANOVA, vs. control virus, **Figure 4G**) following LPS challenge. Vagal SHANK3 knockdown by *Cre* virus treatment also resulted in a significant reduction in survival rate after LPS intervention in *Shank3^f/f^* mice (*p* = 0.0274, vs. control virus, **Figure 4H**). Collectively, these data indicate that SHANK3 in vagal sensory neurons confers protection against LPS-induced hypothermia, systemic inflammation and septic death.

### SHANK3 regulates core temperature and systemic inflammation *via* TRPM2

**Figure 5:**
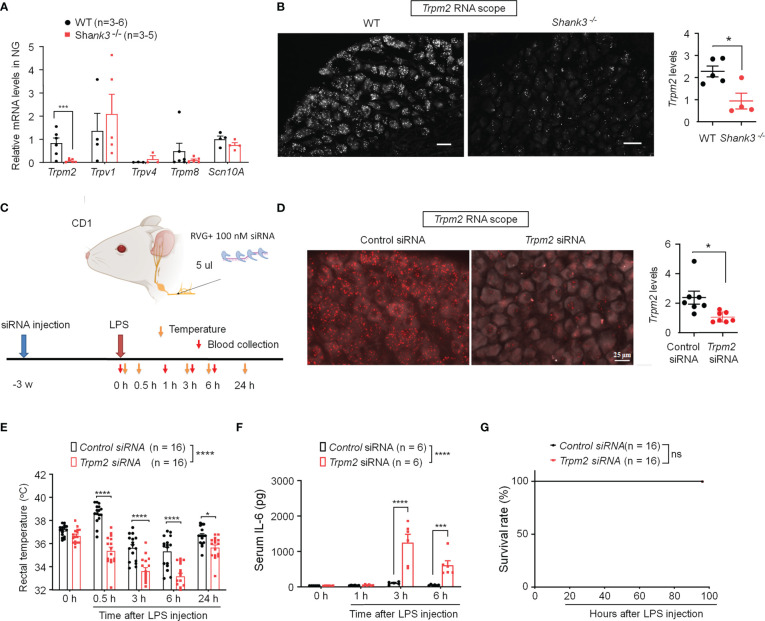
SHANK3 regulates body temperature and inflammation *via* TRPM2 in C57BL/6 mice **(A, B)** and CD1 mice **(C–G)**. **(A)** RT-PCR analysis showing the expression of *Trpm2, Trpv1, Trpv4, Trpm8* and *Scn10A* in NG of WT mice and *Shank3* ^−/−^ mice. **(B)** ISH images showing *Trpm2* mRNA expression in NG neurons of WT mice and *Shank3* ^−/−^ mice. Scale bar, 50 μm. Right, quantification of *Shank3* mRNA levels. ^*^ *p* < 0.05. **(C)** Experimental design for TRPM2 knockdown, LPS injection, body temperature measurement and blood collection in CD1 mice. Peri-neural injection of 100 nM *Trpm2* siRNA was made to knockdown TRPM2 expression in NG neurons. **(D)** ISH images showing *Trpm2* mRNA expression in NG neurons following control siRNA and *Trpm2* siRNA injection. Scale bar, 50 μm. Right, quantification of *Trpm2* mRNA levels. ^*^ *p* < 0.05. **(E)** Time course of rectal temperature after LPS injection (1mg/kg, i.p.) in control siRNA and *Trpm2* siRNA treated mice. **(F)** Serum IL-6 levels and **(G)** survival curves after LPS exposure in control siRNA and *Trpm2* siRNA treated mice. Data are expressed as mean ± SEM and analyzed by unpaired two-tailed *t* test **(B, D)**, two-way ANOVA with Bonferroni *post hoc* test **(E, F)**, and Mantel-Cox test **(G)**. ^*^ *p* < 0.05, ^***^ *p* < 0.001, ^****^ *p* < 0.0001 versus WT mice or mice treated control siRNA. Sample sizes are indicated in brackets. ns, not significant.

TRP channels are thermosensors in peripheral nervous system and detect ambient temperature changes (35, 36). First, we employed quantitative RT-PCR to measure mRNA expressions of *Trpm2, Trpv1, Trpv4, Trpm8* and *Scn10A* (encoding Nav1.8) in NG of WT and GKO mice. Interestingly, we detected a robust reduction in *Trpm2* (*p* = 0.00754) but not *Trpv1, Trpv4, Trpm8* and *Scn10A* mRNA levels in NG of *Shank3* ^−/−^ mice (**Figure 5A**). ISH revealed a broad expression of *Trpm2* mRNA in majority of NG neurons in WT mice, but this expression was significantly lower in *Shank3* ^−/−^ mice (*p* < 0.05, vs. WT, **Figure 5B**).

To further verify the potential role of peripheral TRPM2 in vagal nerve in sepsis-induced hypothermia, *Trpm2* siRNA (100 nM) was employed to knockdown endogenous TRPM2 expression in vagal sensory neurons of CD1 mice through bilateral peri-vagal nerve delivery (**Figure 5C**). ISH detected a significant reduction in *Trpm2* mRNA in NG neurons following the siRNA treatment (*p* = 0.01331, **Figure 5D**). Following LPS challenge, *Trpm2* siRNA treatment exacerbated hypothermia (F (1, 150) = 120.4, *P* < 0.0001, vs. control siRNA, **Figure 5E**) and increased serum IL-6 levels (F (1, 39) = 39.31, *p* < 0.0001, vs. control siRNA, **Figure 5F**), without affecting mortality in CD1 mice (**Figure 5G**). Together, these data indicate that 1) TRPM2 is a downstream event of SHANK3 signaling in NG neurons and 2) loss of TRPM2 in vagal sensory neurons can recapitulate some of the key features of SHANK3 knockdown, including hypothermia and systemic IL-6 increase.

### SHANK2 or TRPV1 deficiency in peripheral sensory neurons fails to alter body temperature homeostasis and systemic inflammation

**Figure 6:**
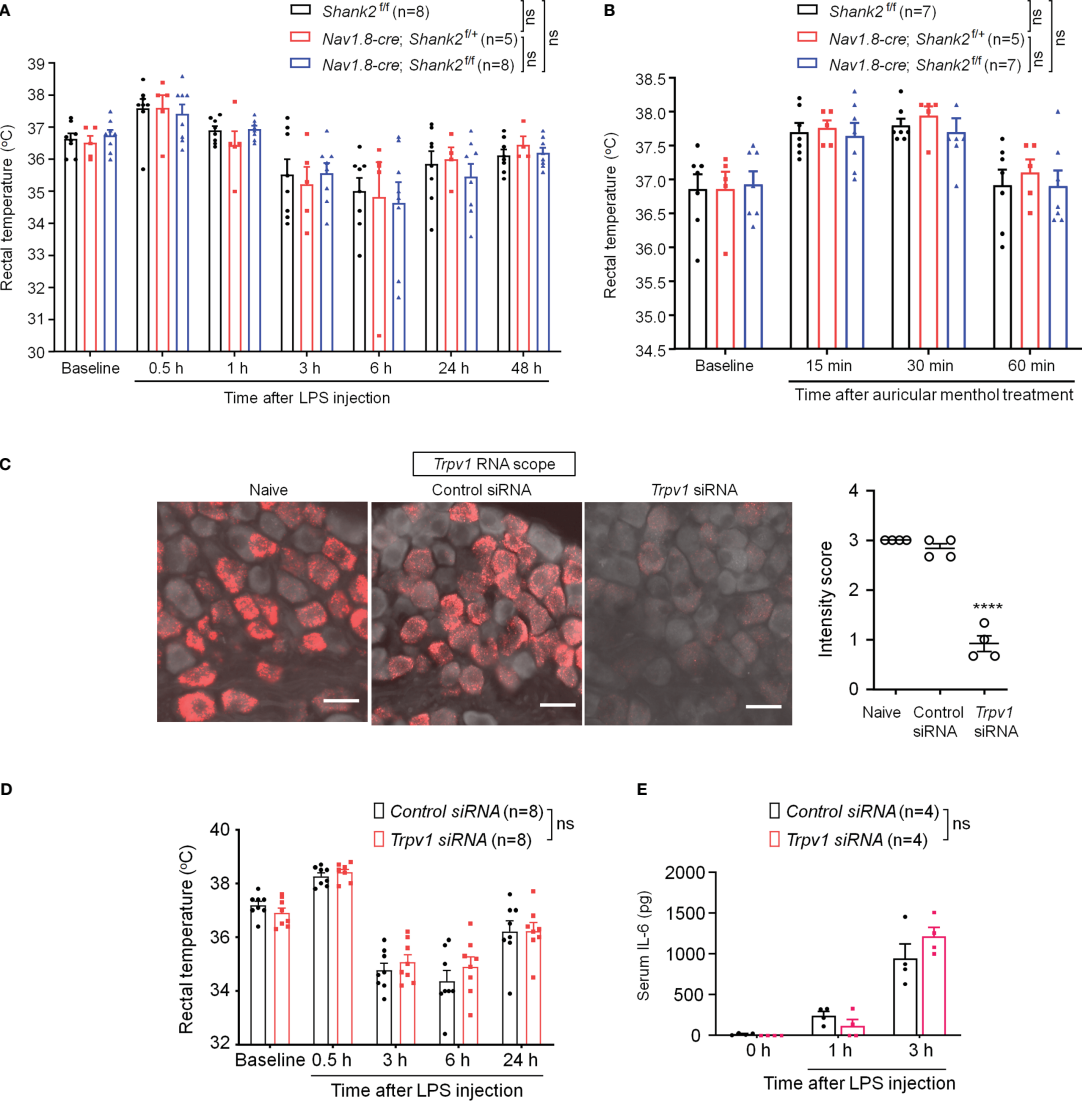
SHANK2 and TRPV1 are dispensable in LPS-induced hypothermia and systemic inflammation in C57BL/6 mice. *Shank2* conditional knockout mice were generated by crossing *Shank2*-floxed mice with *Nav1.8*-*Cre* mice, leading to specific loss of *Shank2* in Nav1.8-expressing sensory neurons. **(A)** Time course of rectal temperature after LPS injection (1mg/kg, i.p.) in *Shank2* ^f/f^ mice, *Nav1.8-cre*; *Shank2* ^f/+^ mice and *Nav1.8-cre*; *Shank2* ^f/f^ mice. **(B)** Time course of rectal temperature after auricular painting of 5% menthol in *Shank2* ^f/f^ mice, *Nav1.8-cre*; *Shank2* ^f/+^ mice and *Nav1.8-cre*; *Shank2* ^f/f^ mice. **(C)** Peri-neural injection of 100 nM *Trpv1* siRNA was made to knockdown TRPV1 expression in vagal sensory neurons in NG. ISH images showing *Trpv1* mRNA (red) expression in NG neurons following control siRNA and *Trpv1* siRNA injection. Scale bar, 50 μm. ****p < 0.0001, unpaired t-test. **(D)** Time course of rectal temperature after LPS injection (1mg/kg, (i) p.) in control siRNA and *Trpv1* siRNA treated mice. **(E)** Serum IL-6 levels after LPS exposure in control siRNA and *Trpv1* siRNA treated mice. Data are expressed as mean ± SEM and analyzed by two-way or one-way ANOVA with Bonferroni *post hoc* test. Sample sizes are indicated in brackets. Not significant, n.s. ****p < 0.0001 versus naive mice.

Dysfunction of SHANK2 protein is also a critical step for the development of autistic-like behaviors (2, 37). We therefore investigated whether SHANK2 is involved in LPS and menthol induced thermal regulation using *Shank2* CKO mice with *Shank2* deletion in Nav1.8^+^ sensory neurons. We did not find significant differences in LPS-induced hypothermia (F (2, 124) = 0.1497, *p* = 0.8611, two-way ANOVA, **Figure 6A**) and survival rate (**Figure S5A**) between littermate control mice (*Shank2^f/f^*) and CKO mice (*Nav1.8-cre; Shank2^f/+^* and *Nav1.8-cre; Shank2^f/f^*). In parallel, we did not observe significant differences in menthol-induced hyperthermia (F (2, 64) = 0.3973, *p* = 0.6738, two-way ANOVA, **Figure 6B**), capsaicin-induced hyperthermia (**Figure S5B**), and anesthesia-induced hypothermia (**Figure S5C**). Thus, *Shank2* in peripheral sensory neurons is not involved in the regulation of body core temperature.

Given a functional link between SHANK3 and TRPV1 in DRG primary sensory neurons and heat sensation (24), we evaluated whether peripheral TRPV1 is important for sepsis-induced hypothermia. *Trpv1* siRNA was employed to knockdown endogenous TRPV1 expression in NG neurons *via* peri-vagal nerve delivery in CD1 mice. ISH detected a substantial reduction in *Trpv1* mRNA in NG neurons following *Trpv1* siRNA (100 nM) injection [F (2, 9) = 115.8, *p* < 0.0001, **Figure 6C**]. Compared to the control siRNA, *Trpv1* siRNA had no significant effects on LPS-induced hypothermia (F (1, 70) = 0.648, *p* = 0.4235, two-way ANOVA, **Figure 6D**) and serum expressions of IL-6 (F (1, 18) = 0.3131, *p* = 0.5827, **Figure 6E**). These findings suggest that TRPV1 in vagal sensory neurons is not required for sepsis-induced hypothermia, systemic inflammation, and mortality.

## Discussion

In this study, using *Shank3* complete knockout mice with deletion of exons 4 to 22 (Δe4-22 *Shank3* **^─/─^**) (8, 24), we have made several interesting findings. First, SHANK3 is broadly expressed in vagal sensory neurons in NG. Second, *Shank3* regulates *Trpm2* expression in vagal sensory neurons. Third, *Shank3* deficiency, but not *Shank2* and *Trpv1* deficiency, exacerbates hypothermia, systemic inflammation (serum IL-6 levels), and mortality in mice, induced by a low dose of lipopolysaccharide (1 mg/kg). Fourth, *Shank3* CKO mice with *Shank3* deficiency in Nav1.8-expressing sensory neurons can recapitulate all these detrimental effects of LPS in GKO mice. Strikingly, selective knockdown of *Shank3* or *Trpm2* in vagal sensory neurons is sufficient to mediate these actions of SHANK3. Our study highlights a novel role of vagal sensory neurons in mediating SHANK3-dependent disorders.

Mutations in the *SHANK3* gene have been recognized as a genetic risk factor for ASD (2). Notably, heterozygous *SHANK3* mutations are typically associated with idiopathic ASD in patients, but heterozygous deletion of *Shank3* gene in mice does not commonly induce ASD-related behavioral deficit (7, 8). Using △e4-22 *Shank3* full knockout strategy, *Shank3* haploinsufficiency was sufficient to cause marked deficits in TRPV1-medicated heat pain. These pain deficits were fully recapitulated in *Shank3* ^+/-^ CKO mice with *Shank3* deficiency in sensory neurons (24). In the present study, *Shank3* ^+/-^ GKO and CKO mice also exhibited significant deficits in LPS-induced hypothermia and systemic inflammation, albeit *Shank3* ^-/-^ GKO and CKO mice had more robust phenotypes. Furthermore, genetically vulnerable *Shank3* ^+/-^ mice, when challenged with LPS to induce an acute inflammatory response, showed robust circuit and behavioral alterations related to ASD (7).

Apart from genetic risk factors, studies also support the hypothesis of immune dysregulation in ASD (3). Especially, IL-6 and IL-17 were upregulated in ASD individuals or animal models of ASD and contribute to autistic behaviors in animal models (3, 13–15). Furthermore, blocking these cytokines with neutralizing antibodies were shown to alleviate autistic behaviors (3, 14). Frequent respiratory infections were found in 57% of ASD patients and regarded as an ASD comorbidity (4). Devastating symptoms were reported in some PMS patients after acute infections or stressful environmental challenges (38). Several clinical studies reported improvements in ASD behaviors following antibiotic treatments (16–18) and immunomodulation (19). Low dose LPS challenge (1-2 mg/kg) induces systemic inflammation (“cytokine storm”) and neuroinflammation and exacerbates ASD symptoms in *Shank3* ^+/-^ mice (7). We found that IL-6 is significantly dysregulated (upregulated) after *Shank3* deficiency, in response to LPS challenge. It will be of great interest to examine the changes of these cytokines in the brain and CSF, which could drive some ASD symptoms.

Accumulating evidence suggests that the vagus system plays a critical role in regulating the gut-brain axis (18, 22, 23), and dysregulation of this axis contributes to ASD (18, 20, 21). Microbial-based therapy has been used to treat social deficits in mouse models of ASD and the vagus nerve is required for this therapeutic effect in *Shank3B* knockout mice (20). VNS is a safe and effective therapy for pediatric patients with drug-resistant epilepsy. There is some evidence that VNS, when performed for epilepsy, may improve behavior in ASD individuals (39). An observational study revealed that VNS not only controlled epilepsy but also improved the autistic behaviors, especially in language, social and self-help (40). It appears that this behavioral improvement occurs independently of VNS’ modulation of seizure (39). Our findings suggest that SHANK3 in vagal sensory neurons may regulate vagal activity. One important vagal regulation is to regulate heart rate. However, this regulatory function of the vagus nerve is impaired after *Shank3* deficiency. This notion is supported by our ECG data demonstrating that auricular VNS reduced heart rate in WT mice but not in *Shank3* KO mice. Vagus nerve regulates the hemostasis of various physiological functions in our body, including body temperature and inflammation. VNS and peripheral nerve stimulation (e.g., acupuncture) confer protection against LPS-induced hypothermia, systemic inflammation, and septic death (22, 41, 42).

Using sensitive RNAscope ISH, we revealed broad expression of *Shank3* mRNA in majority of NG neurons. Importantly, selective knockdown of *Shank3* by siRNA and AAV Cre virus, *via* a perineural delivery approach (29, 30), was sufficient to produce the phenotypes (hypothermia and excessive inflammation) in *Shank3* GKO and CKO mice. To our knowledge this is the first report to demonstrate the function of *Shank3* in vagal sensory neurons. Single-cell RNAseq showed low level expression of *Shank3* in some vagal sensory neurons (43). Earlier study showed that SHANK3 expression in primary sensory neurons regulates thermal pain and heat hyperalgesia (24). As a scaffold protein, SHANK3 interacts with TRPV1 and regulates the surface expression of this heat sensor. Thus, capsaicin-induced cellular responses and pain were largely abolished in mice with *Shank3* deficiency (24). NG sensory neurons also express multiple TRP channels, including TRPV1, TRPA1, TRPM8, and TRPM2 (43). Interestingly, *Shank3* deficiency resulted in reduced expression of *Trpm2*, without changing the expression of *Trpv1*, *Trpv4*, *Trpm8*, and *SCN10A* (encoding Nav1.8). This raises a possibility of transcriptional regulation of *Trpm2* by SHANK3. Interestingly, SHANK3 was found in the nuclei of primary sensory neurons (24). Future studies are needed to investigate how SHANK3 regulates the transcription of *Trpm2*. TRPM2 is a non-selective cation channel. In hypothalamic neurons, TRPM2 channel is a heat sensor. TRPM2 in hypothalamus can limit fever and drive hypothermia (44). TRPM2 is also expressed abundantly in immune cells and is important in inflammatory processes (45). Especially, TRPM2 acts as an oxidant sensor and oxidant sensing by TRPM2 inhibits neutrophil migration and mitigates inflammation (46). We speculate that TRPM2 in vagal sensory neurons could sense the LPS-induced inflammatory oxidants (e.g., ROS). Thus, activation of TRPM2 by stress and oxidants in NG neurons will result in calcium influx and activation of vagal sensory neurons to mediate the anti-inflammatory actions of the VNS. It is noteworthy that basal body temperature under the non-stressful normal conditions does not require *Shank3* and *Trmp2* in vagal sensory neurons. However, SHANK3 or/and TRPM2 play an important role in maintaining body temperature, under stressful conditions with temperature perturbations, such as LPS challenge, VNS by auricular application of menthol and capsaicin, cold environment, and PGE2-induced fever.

Our results also showed that *Shank3* deficiency in GKO and CKO mice or *Shank3* knockdown in vagal sensory neurons lead to increased mortality in response to systemic LPS challenge (1 mg/kg, i.p.). This low-dose LPS challenge produced very low mortality (10%) in WT mice but resulted in 50% mortality in global and conditional *Shank* ^-/-^ mice. This septic death after *Shank3* deficiency may result from a substantial loss of body temperature (hypothermia, <32°C body temperature was closely associated with septic death) and excessive inflammation by cytokine storm. LPS-induced IL-6 upregulation is highly correlated to septic death (28). Among the inflammatory cytokines we measured in this study, only IL-6 is significantly dysregulated after *Shank3* and *Trpm2* deficiency, in response to LPS challenge. A recent study in 2021 was conducted in 9754 autism pupils in Scotland, which is 1.2% out of 787,666 pupils examined. The authors cautiously concluded that the death rate in the current generation of children and young adults with autism is no higher than for other children, in part due to improved diagnosis and care (47). It is noteworthy that ASD children with *SHANK3* mutations exhibit frequent respiratory infections in 57% cases (4). It will be of great interest to study if individuals with *SHANK3* mutations have a higher risk of mortality than ASD individuals with other genetic mutations.

In summary, our findings demonstrate a novel role of SHANK3 in vagal sensory neurons in maintaining body temperature after stress-related perturbations. Furthermore, vagal SHANK3 can limit excessive inflammation after LPS challenge, offering new molecular insights into inflammation dysregulation in some ASD individuals. Finally, we provided a novel link between SHANK3 and TRPM2, an oxidant/stress sensor, in vagal sensory neurons. Recent study connected SHANK to TRPV4 in the Nucleus Accumbens, as TRPV4 inhibition could alleviate autistic behaviors in Shank3^+/-^ mice (7). It remains to be investigated whether impairment of SHANK3-medated vagal modulation is sufficient to drive behavioral changes in ASD, moreover, whether TRPM2 agonist can improve the ASD symptoms.

## Data availability statement

The original contributions presented in the study are included in the article/**Supplementary Material**. Further inquiries can be directed to the corresponding author.

## Ethics statement

The animal study was reviewed and approved by Duke University IACUC.

## Author contributions

LZ, SB and R-RJ designed research; LZ, SB, QH, MM, and XL performed research and analyzed data; LZ, SB, Y-HJ and R-RJ wrote the manuscript and other authors edited the manuscript. All authors contributed to the article and approved the submitted version.

## References

[1] Kaiser T Feng G. Modeling psychiatric disorders for developing effective treatments. Nat Med (2015) 21:979–88. doi: 10.1038/nm.3935 PMC4886231 26340119

[2] Jiang YH Ehlers MD. Modeling autism by SHANK gene mutations in mice. Neuron (2013) 78:8–27. doi: 10.1016/j.neuron.2013.03.016 23583105 PMC3659167

[3] Azhari A Azizan F Esposito G. A systematic review of gut-immune-brain mechanisms in autism spectrum disorder. Dev Psychobiol (2019) 61:752–71. doi: 10.1002/dev.21803 30523646

[4] Liu C Li D Yang H Li H Xu Q Zhou B. Altered striatum centered brain structures in SHANK3 deficient Chinese children with genotype and phenotype profiling. Prog Neurobiol (2021) 200:101985. doi: 10.1016/j.pneurobio.2020.101985 33388374 PMC8572121

[5] Orefice LL. Outside-in: Rethinking the etiology of autism spectrum disorders. Science (2019) 366:45–6. doi: 10.1126/science.aaz3880 31604296

[6] Sarasua SM Boccuto L Sharp JL Dwivedi A Chen CF Rollins JD. Clinical and genomic evaluation of 201 patients with phelan-McDermid syndrome. Hum.Genet (2014) 133:847–59. doi: 10.1007/s00439-014-1423-7 24481935

[7] Tzanoulinou S Musardo S Contestabile A Bariselli S Casarotto G Magrinelli E. Inhibition of Trpv4 rescues circuit and social deficits unmasked by acute inflammatory response in a Shank3 mouse model of autism. Mol Psychiatry (2022) 27:2080–94. doi: 10.1038/s41380-021-01427-0 PMC9126815 35022531

[8] Wang X Bey AL Katz BM Badea A Kim N David LK. Altered mGluR5-homer scaffolds and corticostriatal connectivity in a Shank3 complete knockout model of autism. Nat Commun (2016) 7:11459. doi: 10.1038/ncomms11459 27161151 PMC4866051

[9] Peca J Feliciano C Ting JT Wang W Wells MF Venkatraman TN. Shank3 mutant mice display autistic-like behaviours and striatal dysfunction. Nature (2011) 472:437–42. doi: 10.1038/nature09965 PMC3090611 21423165

[10] Li X Chauhan A Sheikh AM Patil S Chauhan V Li XM. Elevated immune response in the brain of autistic patients. J Neuroimmunol (2009) 207:111–6. doi: 10.1016/j.jneuroim.2008.12.002 PMC2770268 19157572

[11] Jyonouchi H Sun S Le H. Proinflammatory and regulatory cytokine production associated with innate and adaptive immune responses in children with autism spectrum disorders and developmental regression. J Neuroimmunol (2001) 120:170–9. doi: 10.1016/S0165-5728(01)00421-0 11694332

[12] Masi A Quintana DS Glozier N Lloyd AR Hickie IB Guastella AJ. Cytokine aberrations in autism spectrum disorder: A systematic review and meta-analysis. Mol Psychiatry (2015) 20:440–6. doi: 10.1038/mp.2014.59 24934179

[13] Smith SE Li J Garbett K Mirnics K Patterson PH. Maternal immune activation alters fetal brain development through interleukin-6. J Neurosci (2007) 27:10695–702. doi: 10.1523/JNEUROSCI.2178-07.2007 PMC2387067 17913903

[14] Choi GB Yim YS Wong H Kim S Kim H Kim SV. The maternal interleukin-17a pathway in mice promotes autism-like phenotypes in offspring. Science (2016) 351:933–9. doi: 10.1126/science.aad0314 PMC4782964 26822608

[15] Kim S Kim H Yim YS Ha S Atarashi K Tan TG. Maternal gut bacteria promote neurodevelopmental abnormalities in mouse offspring. Nature (2017) 549:528–32. doi: 10.1038/nature23910 PMC5870873 28902840

[16] Sandler RH Finegold SM Bolte ER Buchanan CP Maxwell AP Vaisanen ML. Short-term benefit from oral vancomycin treatment of regressive-onset autism. J Child Neurol (2000) 15:429–35. doi: 10.1177/088307380001500701 10921511

[17] Urbano M Okwara L Manser P Hartmann K Herndon A Deutsch SI. A trial of d-cycloserine to treat stereotypies in older adolescents and young adults with autism spectrum disorder. Clin Neuropharmacol (2014) 37:69–72. doi: 10.1097/WNF.0000000000000033 24824660 PMC4354861

[18] Vuong HE Hsiao EY. Emerging roles for the gut microbiome in autism spectrum disorder. Biol Psychiatry (2017) 81:411–23. doi: 10.1016/j.biopsych.2016.08.024 PMC5285286 27773355

[19] Bey AL Gorman MP Gallentine W Kohlenberg TM Frankovich J Jiang YH. Subacute neuropsychiatric syndrome in girls with SHANK3 mutations responds to immunomodulation. Pediatrics (2020) 145:e20191490. doi: 10.1542/peds.2019-1490 32015180 PMC7802010

[20] Sgritta M Dooling SW Buffington SA Momin EN Francis MB Britton RA. Mechanisms underlying microbial-mediated changes in social behavior in mouse models of autism spectrum disorder. Neuron (2019) 101:246–59.e6. doi: 10.1016/j.neuron.2018.11.018 30522820 PMC6645363

[21] Buffington SA Di Prisco GV Auchtung TA Ajami NJ Petrosino JF Costa-Mattioli M. Microbial reconstitution reverses maternal diet-induced social and synaptic deficits in offspring. Cell (2016) 165:1762–75. doi: 10.1016/j.cell.2016.06.001 PMC5102250 27315483

[22] Tracey KJ. The inflammatory reflex. Nature (2002) 420:853–9. doi: 10.1038/nature01321 12490958

[23] Yu CD Xu QJ Chang RB. Vagal sensory neurons and gut-brain signaling. Curr Opin Neurobiol (2020) 62:133–40. doi: 10.1016/j.conb.2020.03.006 PMC7560965 32380360

[24] Han Q Kim YH Wang X Liu D Zhang ZJ Bey AL. SHANK3 deficiency impairs heat hyperalgesia and TRPV1 signaling in primary sensory neurons. Neuron (2016) 92(6):1279–93. doi: 10.1016/j.neuron.2016.11.007 PMC5182147 27916453

[25] Orefice LL Mosko JR Morency DT Wells MF Tasnim A Mozeika SM. Targeting peripheral somatosensory neurons to improve tactile-related phenotypes in ASD models. Cell (2019) 178:867–86.e24. doi: 10.1016/j.cell.2019.07.024 31398341 PMC6704376

[26] Bey AL Wang X Yan H Kim N Passman RL Yang Y. Brain region-specific disruption of Shank3 in mice reveals a dissociation for cortical and striatal circuits in autism-related behaviors. Transl Psychiatry (2018) 8(1):94. doi: 10.1038/s41398-018-0142-6 29700290 PMC5919902

[27] Agarwal N Offermanns S Kuner R. Conditional gene deletion in primary nociceptive neurons of trigeminal ganglia and dorsal root ganglia. Genesis (2004) 38:122–9. doi: 10.1002/gene.20010 15048809

[28] Bang S Donnelly CR Luo X Toro-Moreno M Tao X Wang Z. Activation of GPR37 in macrophages confers protection against infection-induced sepsis and pain-like behaviour in mice. Nat Commun (2021) 12:1704. doi: 10.1038/s41467-021-21940-8 33731716 PMC7969930

[29] Berta T Park CK Xu ZZ Xie RG Liu T Lu N. Extracellular caspase-6 drives murine inflammatory pain *via* microglial TNF-alpha secretion. J Clin Invest (2014) 124:1173–86. doi: 10.1172/JCI72230 PMC3934175 24531553

[30] Kumar P Wu H McBride JL Jung KE Kim MH Davidson BL. Transvascular delivery of small interfering RNA to the central nervous system. Nature (2007) 448:39–43. doi: 10.1038/nature05901 17572664

[31] Zanos TP Silverman HA Levy T Tsaava T Battinelli E Lorraine PW. Identification of cytokine-specific sensory neural signals by decoding murine vagus nerve activity. Proc Natl Acad Sci USA (2018) 115:E4843–52. doi: 10.1073/pnas.1719083115 PMC6003492 29735654

[32] Mishra V Gautier NM Glasscock E. Simultaneous video-EEG-ECG monitoring to identify neurocardiac dysfunction in mouse models of epilepsy. J Vis Exp (2018) (131):57300. doi: 10.3791/57300-v 29443088 PMC5912255

[33] Stirling LC Forlani G Baker MD Wood JN Matthews EA Dickenson AH. Nociceptor-specific gene deletion using heterozygous NaV1.8-cre recombinase mice. Pain (2005) 113:27–36. doi: 10.1016/j.pain.2004.08.015 15621361

[34] Shields SD Ahn HS Yang Y Han C Seal RP Wood JN. Nav1.8 expression is not restricted to nociceptors in mouse peripheral nervous system. Pain (2012) 153:2017–30. doi: 10.1016/j.pain.2012.04.022 22703890

[35] Vriens J Nilius B Voets T. Peripheral thermosensation in mammals. Nat Rev Neurosci (2014) 15:573–89. doi: 10.1038/nrn3784 25053448

[36] Patapoutian A Peier AM Story GM Viswanath V. ThermoTRP channels and beyond: Mechanisms of temperature sensation. Nat Rev Neurosci (2003) 4:529–39. doi: 10.1038/nrn1141 12838328

[37] Pappas AL Bey AL Wang X Rossi M Kim YH Yan H. Deficiency of Shank2 causes mania-like behavior that responds to mood stabilizers. JCI Insight (2017) 2:e92052. doi: 10.1172/jci.insight.92052 29046483 PMC5846902

[38] Kolevzon A Delaby E Berry-Kravis E Buxbaum JD Betancur C. Neuropsychiatric decompensation in adolescents and adults with phelan-McDermid syndrome: a systematic review of the literature. Mol Autism (2019) 10:50. doi: 10.1186/s13229-019-0291-3 31879555 PMC6930682

[39] van Hoorn A Carpenter T Oak K Laugharne R Ring H Shankar R. Neuromodulation of autism spectrum disorders using vagal nerve stimulation. J Clin Neurosci (2019) 63:8–12. doi: 10.1016/j.jocn.2019.01.042 30732986

[40] Wang Z Yuan X Zhang Q Wen J Cheng T Qin X. Effects of stable vagus nerve stimulation efficacy on autistic behaviors in ten pediatric patients with drug resistant epilepsy: An observational study. Front Pediatr (2022) 10:846301. doi: 10.3389/fped.2022.846301 35311037 PMC8924444

[41] Torres-Rosas R Yehia G Pena G Mishra P del Rocio Thompson-Bonilla M Moreno-Eutimio MA. Dopamine mediates vagal modulation of the immune system by electroacupuncture. Nat Med (2014) 20:291–5. doi: 10.1038/nm.3479 PMC3949155 24562381

[42] Liu S Wang Z Su Y Qi L Yang W Fu M. A neuroanatomical basis for electroacupuncture to drive the vagal-adrenal axis. Nature (2021) 598:641–5. doi: 10.1038/s41586-021-04001-4 PMC9178665 34646018

[43] Kupari J Haring M Agirre E Castelo-Branco G Ernfors P. An atlas of vagal sensory neurons and their molecular specialization. Cell Rep (2019) 27:2508–23.e4. doi: 10.1016/j.celrep.2019.04.096 31116992 PMC6533201

[44] Song K Wang H Kamm GB Pohle J Reis FC Heppenstall P. The TRPM2 channel is a hypothalamic heat sensor that limits fever and can drive hypothermia. Science (2016) 353:1393–8. doi: 10.1126/science.aaf7537 PMC7612276 27562954

[45] Haraguchi K Kawamoto A Isami K Maeda S Kusano A Asakura K. TRPM2 contributes to inflammatory and neuropathic pain through the aggravation of pronociceptive inflammatory responses in mice. J Neurosci (2012) 32:3931–41. doi: 10.1523/JNEUROSCI.4703-11.2012 PMC6703465 22423113

[46] Wang G Cao L Liu X Sieracki NA Di A Wen X. Oxidant sensing by TRPM2 inhibits neutrophil migration and mitigates inflammation. Dev Cell (2016) 38:453–62. doi: 10.1016/j.devcel.2016.07.014 PMC5455786 27569419

[47] Smith GS Fleming M Kinnear D Henderson A Pell JP Melville C. Mortality in 787,666 school pupils with and without autism: A cohort study. Autism (2021) 25:300–4. doi: 10.1177/1362361320944037 PMC7812511 32830516

