# Supplementary material for: SHANK3 in vagal sensory neurons regulates body temperature, systemic inflammation, and sepsis
Source: Front Immunol. 2023 Feb 8;14:1124356. doi: 10.3389/fimmu.2023.1124356 (PMC9944123; doi:10.3389/fimmu.2023.1124356)
Supplement: Supplementary file 1 [file DataSheet_1.pdf]

SHANK3 in Vagal Sensory Neurons Regulates Body Temperature, Systemic Inflammation, and Sepsis

Linlin Zhang, Sangsu Bang, Qianru He, Megumi Matsuda, Xin Luo, Yong-Hui Jiang, and Ru-Rong Ji

Supplemental Figures 1-6

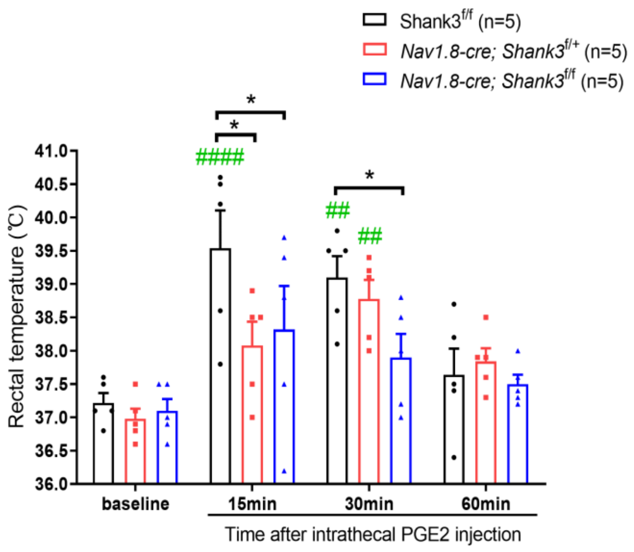

Figure S1: PGE$_2$-induced hyperthermia is abrogated after *Shank3* deficiency in sensory neurons of C57BL/6 mice.

Time course of rectal temperature after intrathecal (i.t.) injection of PGE$_2$ (200 ng) in *Shank3*$^{f/f}$ mice, *Nav1.8-cre*; *Shank3*$^{f/+}$ mice and *Nav1.8-cre*; *Shank3*$^{f/f}$ mice. *Shank3* CKO mice were generated by crossing *Shank3*-floxed mice with *Nav1.8-Cre* mice, leading to specific loss of *Shank3* in Nav1.8-expressing sensory neurons. Data are expressed as mean ± SEM and analyzed by two-way ANOVA with Bonferroni *post hoc* test. $^{\#\#}p < 0.01$, $^{\#\#\#\#}p < 0.0001$ versus baseline. $^{*}p < 0.05$ versus *Shank3*$^{f/f}$ mice. Sample sizes are indicated in brackets.

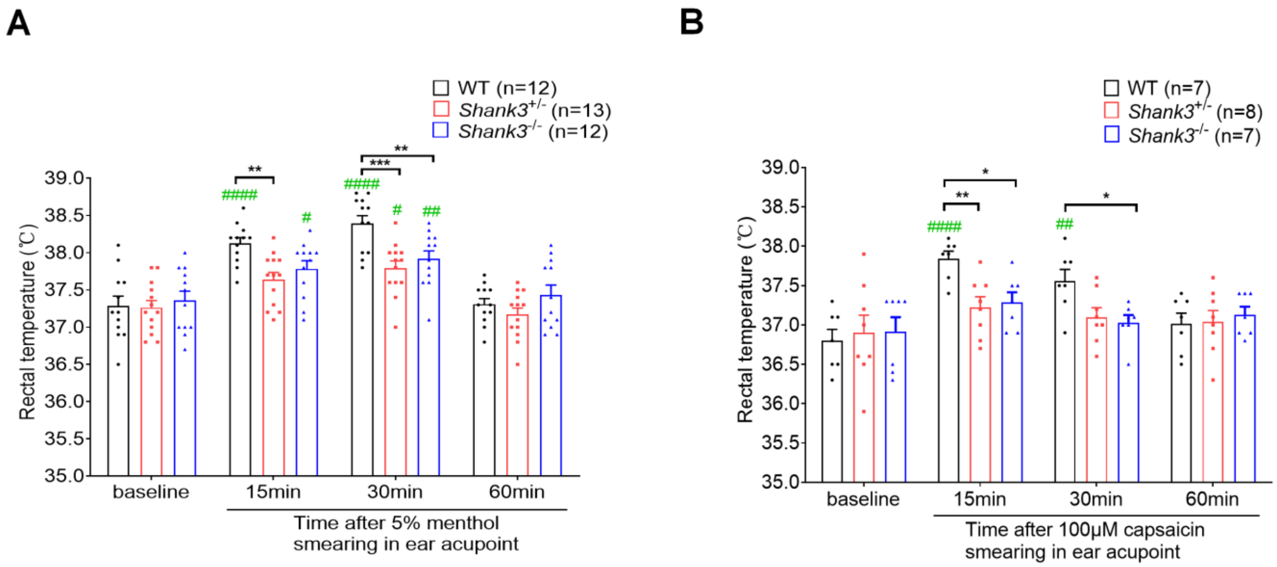

Figure S2: Menthol and capsaicin induced hyperthermia in WT and *Shank3* C57BL/6 GKO mice.

(A) Time course of rectal temperature after VNS by auricular painting of 5% menthol in Wild-type (WT) mice, heterozygous (*Shank3*$^{+/-}$) mice and homozygous (*Shank3*$^{-/-}$) mice. (B) Time course of rectal temperature after VNS by auricular painting of 100 µM capsaicin in three genotypes. Data are expressed as mean ± SEM and analyzed by two-way ANOVA with Bonferroni *post hoc* comparisons. $^{\#}p < 0.01$, $^{\#\#} p< 0.01$, $^{\#\#\#\#}p < 0.001$ versus baseline. $^{*}p < 0.05$, $^{**}p < 0.01$, $^{***}p < 0.001$ versus WT mice. Sample sizes are indicated in brackets.

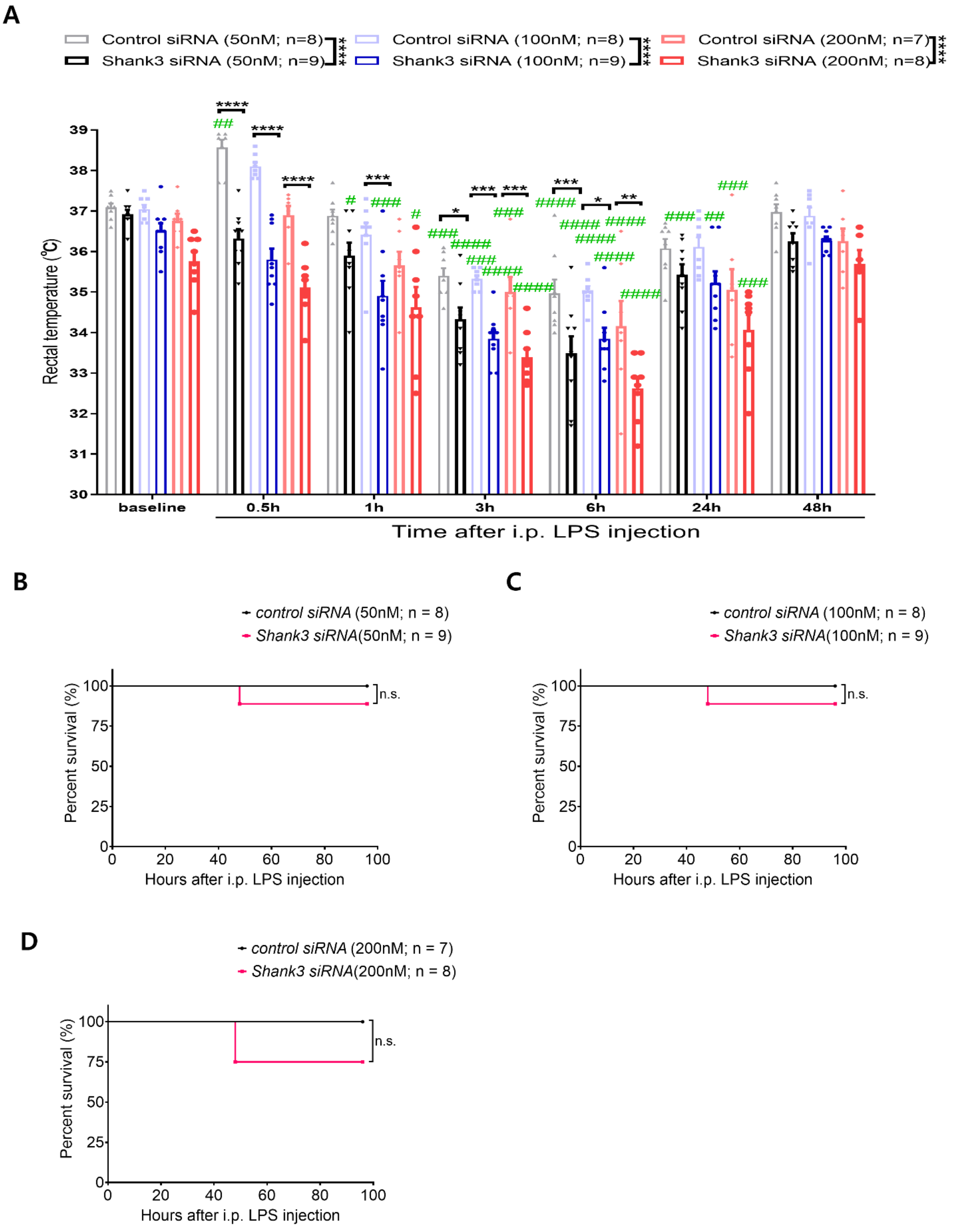

Figure S3: Effects of *Shank3* siRNA with different concentrations on LPS-induced hypothermia and mortality in CD1 mice.

(A) Time course of rectal temperature after LPS injection (1mg/kg, i.p.) in control siRNA and *Shank3* siRNA treated mice. (B-D) Survival curves after LPS exposure in mice treated with control siRNA and *Shank3* siRNA with different concentrations. Different concentrations (50 nM, 100 nM and 200 nM) of *Shank3* siRNA (mixed with RVG peptide, 1:10) were given by peri-neural injections to knockdown SHANK3 expression in vagal sensory neurons in NG in CD1 mice. Data are expressed as mean ± SEM and analyzed by two-way ANOVA with Bonferroni *post hoc* test (A), and Mantel-Cox test (B-D). $^{\#}p < 0.05$, $^{\#\#}p < 0.01$, $^{\#\#\#}p < 0.001$, $^{\#\#\#\#}p < 0.0001$ versus baseline. $^{*}p < 0.05$, $^{**}p < 0.01$, $^{***}p < 0.001$, $^{****}p < 0.0001$ versus mice treated control siRNA. Sample sizes are indicated in brackets. ns, not significant.

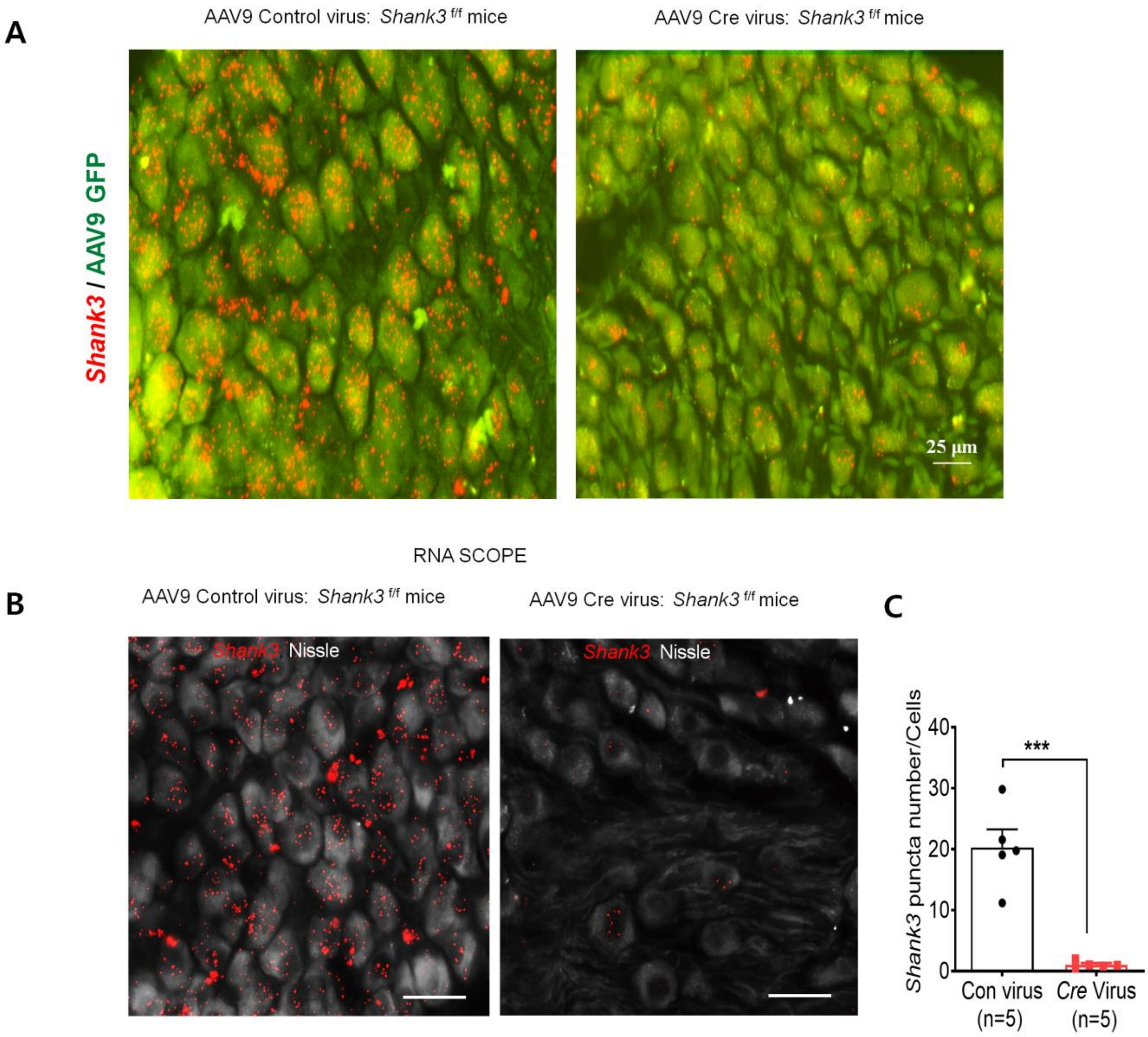

Figure S4: Peri-neural injection of Cre-AAV9 causes *Shank3* knockdown in GFP-expressing NG neurons of *Shank3* $^{f/f}$ C57BL/6 mice.

(A and B) ISH images showing *Shank3* mRNA (red, A and B) and GFP (green, A) expression in NG vagal sensory neurons following peri-neural injection of AAV9 control virus and AAV9 *Cre* virus. Scale bar, 25 µm. (C) Quantification of the number of *Shank3* puncta per neurons in NG. AAV9 virus containing *cre* was delivered by peri-neural injection to knockdown SHANK3 expression in NG vagal sensory neurons in *Shank3*$^{f/f}$ mice. Data are expressed as mean ± SEM and analyzed by unpaired two-tailed *t* test. $^{***}p < 0.001$ vs. control virus. Sample sizes are indicated in brackets.

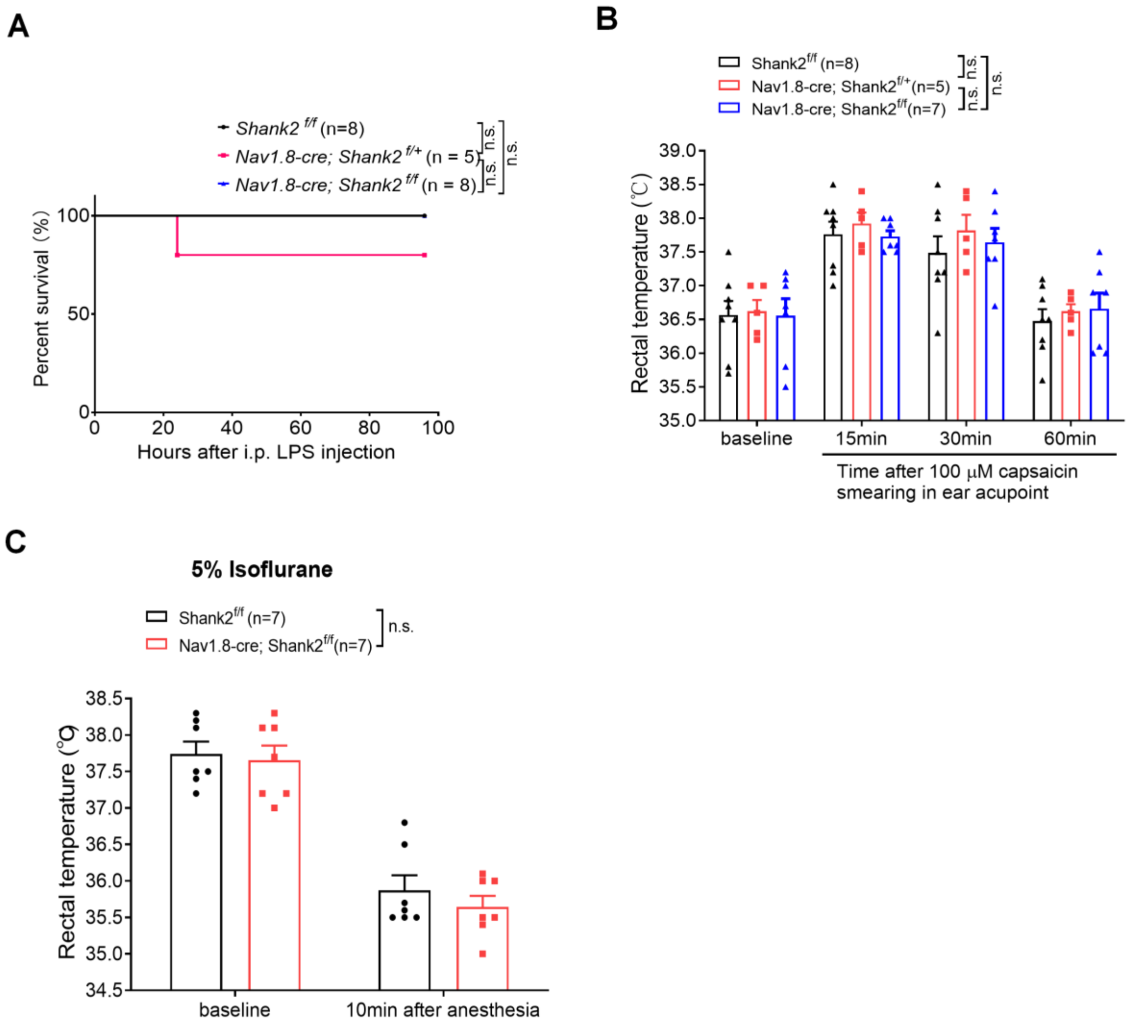

Figure S5: *Shank2* deficiency in sensory neurons has no significant contribution to LPS-induced death and capsaicin and isoflurane induced body temperature perturbations in C57BL/6 mice.

(A) Survival curves after LPS injection (1 mg/kg, i.p.) in *Shank2*$^{f/f}$ mice, *Nav1.8-cre*; *Shank2*$^{f/+}$ mice and *Nav1.8-cre*; *Shank2*$^{f/f}$ mice. (B) Time course of rectal temperature after auricular VNS with 100 μM capsaicin painting in three genotypes. (C) Time course of rectal temperature after brief anesthesia to isoflurane in two genotypes. Data are expressed as mean ± SEM and analyzed by Mantel-Cox test (A), two-way ANOVA with Bonferroni *post hoc* test (B, C). Sample sizes are indicated in brackets. ns, not significant.

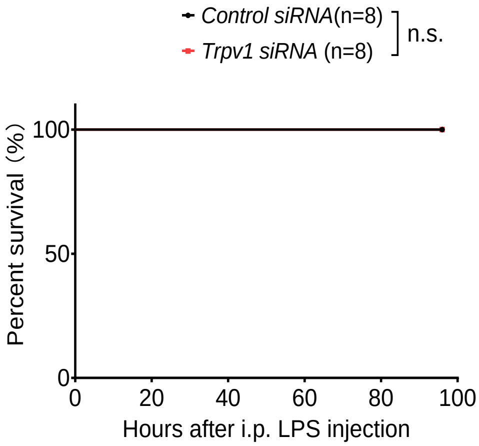

Figure S6: Effects of vagal *Trpv1* siRNA knockdown on LPS-induced mortality in CD1 mice.

Survival curve shows no effects of *Trpv1* siRNA on LPS-induced mortality. Peri-neural injection was conducted by delivery of 100 nM of *Trpv1* siRNA (5 □l, mixed with RVG peptide, 1:10) to knockdown TRPV1 expression in NG vagal sensory neurons in CD1 mice. Data are analyzed by Mantel-Cox test. ns, not significant.
